# The many facets of immunometabolism

**DOI:** 10.1371/journal.pbio.3003641

**Published:** 2026-01-27

**Authors:** Joanna Clarke, Claudio Mauro

**Affiliations:** 1 Public Library of Science, San Francisco, California, United States of America and Cambridge, United Kingdom; 2 College of Medicine and Health, University of Birmingham, Birmingham, United Kingdom

## Abstract

Immunometabolism describes more than just metabolic shifts in immune cells. A new collection of articles shines a light on the many facets of immunometabolism, exploring the effects of molecular, cellular, and systemic metabolic mechanisms in health and disease.

Twenty-five years or so ago, immunologists began to explore how the key enzymatic reactions of cellular metabolism could affect the immune response, in terms of immune cell differentiation, cytotoxicity, helper functions, and cytokine production, giving rise to the field of “immunometabolism”. The core metabolic reactions of a cell, from glycolysis to oxidative phosphorylation, were now being seen as drivers of specialized responses in immune cells. However, the initial studies were often carried out in murine cells or model systems which, although revealing certain metabolic shift paradigms (such as the reliance of pro-inflammatory macrophages on glycolysis or of regulatory T cells on oxidative phosphorylation), often produced results that were different from what takes place in human physiology and pathology [1–3]. The field soon began to move towards more pathophysiologic models, using human cells and tissues from healthy donors or patients, and culture conditions that are more similar to those in the human body. These efforts led to the discovery of metabolic shifts that take place in immune cells in a plethora of diseases, from cancer to obesity, diabetes, and infectious disease [4–6]; for example, the metabolic requirements of checkpoint blockade (e.g., during PD-1–PD-L1 interactions [7]), which has important implications for immune checkpoint blockade therapy for cancer and autoimmune diseases.

One area in which immunometabolism has initially moved more slowly, but is now catching up, is the effect of alterations in systemic metabolism (such as occurs in obesity, type 2 diabetes, cardiovascular diseases, and aging) on immune cells and, in turn, the role of these cells in disease outcomes. Indeed, although classically viewed as metabolic changes that occur in immune cells in response to different stimuli, immunometabolism can also encompass the effects of systemic metabolic changes on immunity, and vice versa. In this collection, we feature articles highlighting these aspects of the field. Yun Sok Lee investigates the process of metaflammation, the chronic low-grade inflammatory response that occurs in metabolic diseases such as obesity and type 2 diabetes, exploring how systemic changes in metabolism can affect tissue-resident immune cells [8]. Intriguingly, he lays out the evidence supporting an unexpected beneficial role for inflammation during the early stages of obesity, and explains how this helpful inflammatory response can later become detrimental.

Also looking at systemic metabolic effects, Griffin Gowdy and Alice Prince discuss how released immunometabolites can shape bacterial infections [9]. Metabolites such as itaconate and succinate have long been characterized as key regulators of immune responses to infections and cancer [2,4]. In their Essay, Gowdy and Prince look at the role of such metabolites (produced by the pathogen and by the host) in both driving and combating bacterial infections. They focus on pathogens that infect the lungs, both because of the interesting immune dynamics at play in this organ system, and due to the global health relevance of understanding and treating lung infections.

A long-standing goal of the immunometabolism field has been to translate findings from the lab into the clinic, either by targeting metabolism at a systemic or cellular level, or by harnessing metabolites therapeutically [4]. In their Perspective, Nicole Chapman and Hongbo Chi advocate for the use of systems biology approaches in immunometabolism research as discovery tools for new therapeutic targets [10]. They suggest that combining single-cell omics technologies with state-of-the-art CRISPR screening techniques could help to answer questions about emerging concepts in immunometabolism, such as nutrient signaling, metabolic heterogeneity, and intercellular metabolic communication. On a more holistic level, Guang Sheng Ling and colleagues suggest a two-pronged approach to targeting immunometabolism therapeutically through pharmaceutical and lifestyle interventions [11]. Their Essay focuses on this concept in cancer, where evidence suggests that lifestyle changes that target immunometabolism by altering diet, exercise, stress levels, and the gut microbiome could be beneficial to those undergoing more traditional therapy.

The field is clearly moving towards trying to address real medical problems using the knowledge we have gained over the past few decades of how immune metabolic pathways govern and guide the immune response. Understanding the interplay between systemic metabolism and the immune response will be crucial if we want to find ways to intervene in conditions with altered metabolism, ranging from obesity to cancer, as well as in aging. By broadening out our investigations to cover every facet of immunometabolism, we can hope to bring the promise of targeting metabolic pathways into the clinic.
